# Autosomal Recessive Cerebellar Atrophy and Spastic Ataxia in Patients With Pathogenic Biallelic Variants in *GEMIN5*

**DOI:** 10.3389/fcell.2022.783762

**Published:** 2022-02-28

**Authors:** Deepa S. Rajan, Sukhleen Kour, Tyler R. Fortuna, Margot A. Cousin, Sarah S. Barnett, Zhiyv Niu, Dusica Babovic-Vuksanovic, Eric W. Klee, Brian Kirmse, Micheil Innes, Siri Lynne Rydning, Kaja K. Selmer, Magnus Dehli Vigeland, Anne Kjersti Erichsen, Andrea H. Nemeth, Francisca Millan, Catherine DeVile, Katherine Fawcett, Adrien Legendre, David Sims, Ricardo Parolin Schnekenberg, Lydie Burglen, Sandra Mercier, Somayeh Bakhtiari, Rosario Francisco-Velilla, Azman Embarc-Buh, Encarnacion Martinez-Salas, Kristen Wigby, Jerica Lenberg, Jennifer R. Friedman, Michael C. Kruer, Udai Bhan Pandey

**Affiliations:** ^1^ Department of Pediatrics, Children’s Hospital of Pittsburgh, University of Pittsburgh Medical Center, Pittsburgh, PA, United States; ^2^ Department of Center for Individualized Medicine, Mayo Clinic, Rochester, MN, United States; ^3^ Department of Quantitative Health Sciences, Mayo Clinic, Rochester, MN, United States; ^4^ Department of Laboratory Medicine and Pathology, Mayo Clinic, Rochester, MN, United States; ^5^ Department of Clinical Genomics, Mayo Clinic, Rochester, MN, United States; ^6^ Division of Genetics, University of Mississippi Medical Center, Jackson, MS, United States; ^7^ Department of Medical Genetics and Alberta Children's Hospital Research Institute, Cumming School of Medicine, University of Calgary, Calgary, AB, Canada; ^8^ Department of Neurology, Oslo University Hospital, Oslo, Norway; ^9^ Department of Research and Innovation, Division of Clinical Neuroscience, Oslo University Hospital and the University of Oslo, Oslo, Norway; ^10^ Department of Medical Genetics, Oslo University Hospital, and Institute of Clinical Medicine, University of Oslo, Oslo, Norway; ^11^ Department of Ophthalmology, Oslo University Hospital, Oslo, Norway; ^12^ Nuffield Department of Clinical Neurosciences, University of Oxford, Oxford, United Kingdom; ^13^ GeneDx, Gaithersburg, MD, United States; ^14^ Great Ormond Street Hospital, London, United Kingdom; ^15^ Weatherall Institute of Molecular Medicine, University of Oxford, Oxford, United Kingdom; ^16^ Department of Health Sciences, University of Leicester, Leicester, United Kingdom; ^17^ Laboratoire de biologie médicale multisites Seqoia—FMG2025, Paris, France; ^18^ Centre de Référence des Malformations et Maladies Congénitales du Cervelet et Laboratoire de Neurogénétique Moléculaire, Département de Génétique, AP-HP. Sorbonne Université, Hôpital Trousseau, Paris, France; ^19^ Developmental Brain Disorders Laboratory, Imagine Institute, INSERM UMR 1163, Paris, France; ^20^ Barrow Neurological Institute, Phoenix Children’s Hospital, Phoenix, AZ, United States; ^21^ Departments of Child Health, Neurology, Cellular and Molecular Medicine and Program in Genetics, University of Arizona College of Medicine—Phoenix, Phoenix, AZ, United States; ^22^ Centro de Biologia Molecular Severo Ochoa, CSIC-UAM, Madrid, Spain; ^23^ Department of Neurosciences, University of California San Diego, San Diego, CA, United States; ^24^ Department of Pediatrics, University of California San Diego, San Diego, CA, United States; ^25^ Rady Children’s Institute for Genomic Medicine, San Diego, CA, United States; ^26^ CHU Nantes, Service de génétique médicale, Centre de Référence Anomalies du Développement et Syndromes Malformatifs, Nantes, France; ^27^ Nantes Université, CNRS, INSERM, l’institut du thorax, Nantes, France

**Keywords:** Gemin5, ataxia, cerebellar atrophy, developmental delay, neurodegeneration, cell death, development

## Abstract

The hereditary ataxias are a heterogenous group of disorders with an increasing number of causative genes being described. Due to the clinical and genetic heterogeneity seen in these conditions, the majority of such individuals endure a diagnostic odyssey or remain undiagnosed. Defining the molecular etiology can bring insights into the responsible molecular pathways and eventually the identification of therapeutic targets. Here, we describe the identification of biallelic variants in the *GEMIN5* gene among seven unrelated families with nine affected individuals presenting with spastic ataxia and cerebellar atrophy. GEMIN5, an RNA-binding protein, has been shown to regulate transcription and translation machinery. GEMIN5 is a component of small nuclear ribonucleoprotein (snRNP) complexes and helps in the assembly of the spliceosome complexes. We found that biallelic *GEMIN5* variants cause structural abnormalities in the encoded protein and reduce expression of snRNP complex proteins in patient cells compared with unaffected controls. Finally, knocking out endogenous *Gemin5* in mice caused early embryonic lethality, suggesting that Gemin5 expression is crucial for normal development. Our work further expands on the phenotypic spectrum associated with *GEMIN5-*related disease and implicates the role of GEMIN5 among patients with spastic ataxia, cerebellar atrophy, and motor predominant developmental delay.

## Introduction

Hereditary ataxias are a heterogenous group of neurological disorders affecting individuals of all age groups, and patients present with a disturbance in movement coordination. While cerebellar abnormalities are usually implicated, lesions elsewhere in the neuroaxis can lead to gait abnormalities that mimic the clinical phenotype. In addition, it is well recognized that many of the autosomal recessive cerebellar ataxias can present as spastic ataxias with pyramidal signs (Ruano et al., 2014). While traditionally these were clinically distinct entities, a spectrum of diseases ranging in clinical presentation from pure cerebellar ataxias to spastic paraplegias have been observed more recently (Synofzik and Schüle, 2017). The genetic causes of hereditary cerebellar ataxias and hereditary spastic ataxias are heterogenous, and often next-generation sequencing technologies are used to establish a molecular diagnosis (Németh et al., 2013). To date, more than 150 genes (OMIM categories include: 45 genes as autosomal dominant spinocerebellar ataxias, 29 autosomal recessive spinocerebellar ataxias, 8 spastic ataxias, and 73 spastic paraplegias) have been implicated in the ataxia paraplegia spectrum of diseases (McKusick, 2007). The identification of the molecular diagnosis in these patients is the first step toward understanding the molecular mechanism and is crucial for developing effective targeted therapeutic strategies.

GEMIN5 is an RNA-binding protein involved in regulating multiple aspects of transcriptional and translational processes (Francisco-Velilla et al., 2020; Martinez-Salas et al., 2020; Moreno-Morcillo et al., 2020). The GEMIN5 protein has 13 tryptophan–aspartic acid dipeptides (WD domains) at the N-terminus, followed by a dimerization domain, and a noncanonical RNA-binding site (RBS1) domain (Francisco-Velilla et al., 2020; Martinez-Salas et al., 2020; Moreno-Morcillo et al., 2020). GEMIN5 is predominantly a cytoplasmic protein with sparse expression in the nucleus, suggesting that this protein might be involved in regulating functions in both cellular compartments. The WD domain of GEMIN5 assists in the biogenesis of small nuclear ribonucleoproteins (snRNPs), the building blocks of splicing machinery, and aids in the formation of the snRNP complex along with the other complex proteins (SMN, Gemin2-8 and Unrip) (Lozano et al., 2018; Francisco-Velilla et al., 2020). Moreover, the noncanonical RNA-binding sites (RBS), located on the C-terminus, promote GEMIN5 to interact with various mRNAs and regulate their translation. Importantly, mutations affecting the survival motor neuron (SMN) protein have been linked with a neuromuscular disorder, spinal muscular atrophy (SMA), which has been linked with disruption of snRNP complex assembly (Melki et al., 1994; Lefebvre et al., 1995; Lefebvre et al., 1997; Burlet et al., 1998; Pellizzoni et al., 2002). The GEMIN5 protein has numerous functions as it incorporates into cytoplasmic stress granules, binds to ribosomes, and regulates global translation and SMN expression.

Here, we define a novel neurodevelopmental ataxia syndrome in patients with autosomal recessive variants in the *GEMIN5* gene. We provide characterization of their neurological phenotype to include a spectrum of the cerebellar ataxia and spastic paraplegia phenotypes among seven unrelated families with nine affected individuals. We employ computational biology, biochemistry, and mouse genetics to examine the functional consequences of perturbing GEMIN5 protein *in vitro* and *in vivo.* Our data suggest that loss-of-function of GEMIN5 is detrimental in human patient cells and in mice.

## Methods

### Patient Recruitment

*GEMIN5* variants were identified in probands by whole-exome sequencing in clinical diagnostic settings at different sites. We found a subset of our *GEMIN5* patients through GeneMatcher (Wang et al., 2007). All patients were evaluated by a neurologist or geneticist at their respective referral centers. We reviewed the clinical information, neurological symptoms, examination, and radiological studies including brain MRI and other clinical evaluations in each patient. All patient information was deidentified. Informed consent was obtained from patients for publication at each site per local institution requirements by the authors.

### Genetic Studies and Variant Assessment

Whole-exome sequencing (WES) was performed at different genetic centers using next-generation sequencing techniques, and all variants were confirmed *via* Sanger sequencing with standard methods.

Family 1: The trio was sequenced at the Yale Center for Genome Analysis (YCGA). Genomic DNA was captured using an IDT xGen exome kit followed by Illumina DNA sequencing. WES data were processed using two independent pipelines at the Yale School of Medicine and Phoenix Children’s Hospital. At each site, sequence reads were mapped to the reference genome (GRCh37) with BWA-MEM and further processed using GATK Best Practice workflows, which include duplication marking, indel realignment, and base quality recalibration. Single-nucleotide variants and small indels were called with GATK HaplotypeCaller and annotated using ANNOVAR, dbSNP (v138), 1000 Genomes (August 2015), NHLBI Exome Variant Server (EVS), and the Exome Aggregation Consortium v3 (ExAC). Rare deleterious missense variants and LOF variants (stop-gain, stop-loss, frameshift insertions/deletions, canonical splice site, and start-loss) were selected. MetaSVM and Combined Annotation Dependent Deletion (CADD v1.3) algorithms were used to predict deleteriousness of missense variants (MetaSVM-deleterious or CADD ≥20). Family 2: The trio was analyzed at the Genomic Sequencing Platform Seqoia (Paris) as follows: preparation of the libraries using NEBNext® Ultra II End repair/A-tailing module and Ligation module (New England Biolabs®), whole-genome sequencing on a NovaSeq 6000® (Illumina®) using 2 × 150 paired-end sequencing, high-quality reads mapping against the human reference genome (hg38), variant calling with GATK4 v4.1.7.0 (Broad Institute), and annotation with SnpEff (4.3t) and SnpSift (4.3t). Family 3: Extensive metabolic testing, microarray, spinocerebellar ataxia panel, and Friedrich’s ataxia panel were negative. Exome revealed GEMIN5: Chr 5 c.3046 C > T p. Arg 1016cys, c. 1452 dup p. Met485HisfsTer27. Family 4: The exome was done as per methodology described previously (Parolin Schnekenberg et al., 2015). Patients were recruited, and consent for participation in the study was obtained according to the Declaration of Helsinki and approved by the Central Oxford Research Ethics Committee and the Research and Development Department of the Oxford Radcliffe Hospitals NHS Trust, Oxford. All patients or their parents provided written consent for the study.

Family 5: Clinical exome sequencing was performed as described previously (Cousin et al., 2019). The proband and his parents provided written informed consent to a study approved by the Mayo Clinic Institutional Review Board. Family 5 sequence reads were mapped to the reference genome (GRCh37) with BWA-MEM and further processed using GATK Best Practice workflows, which include duplication marking, indel realignment, and base quality recalibration. Single-nucleotide variants and small indels were called with GATK HaplotypeCaller. The compound heterozygous variants originally identified and Sanger confirmed by the clinical laboratory for Family 5 were called with high confidence using the workflow implemented for Family 1. Family 6: Exome sequencing was done as per previously described protocol (Guillen Sacoto et al., 2020; Kour et al., 2021). All the variants are annotated by using the *GEMIN5* NP_056280.2 reference transcript in GnomAD and the other databases to estimate the allelic frequency.

Family 7: Whole-exome sequencing (WES) was performed in the two affected siblings, using DNA from peripheral blood leukocytes. The exome was enriched using the SureSelectXT V5 exome kit (Agilent, Böblingen, Germany) and sequenced on a HiSeq2500 sequencer (Illumina, San Diego, CA, United States). High-quality reads were mapped against the human reference genome (hg19), and variants were called following the Genome Analysis Tool Kit version 3.3.0 best practice recommendations (McKenna et al., 2010; Ma et al., 2013). After annotation with Annovar, filtration and downstream analysis was done with FILTUS (Vigeland et al., 2016). The identified *GEMIN5* variants were validated by Sanger sequencing.

The damaging index of *GEMIN5* variants was determined by using various *in silico* prediction tools, such as Polyphen2, Provean, SNAP2, MUpro, PhD SNP, and SIFT (Cheng et al., 2006; Ng and Henikoff, 2006; Bromberg and Rost, 2007; Mi et al., 2010; Choi et al., 2012; Sim et al., 2012). Candidate variants were validated by Sanger sequencing and tested for cosegregation in all family members whenever samples were available (Supplementary Figure S1).

All the variants were annotated by using the GEMIN5 NP_056280.2 reference transcript to estimate the allelic frequency in GnomAD and the other databases. The effect of GEMIN5 variants on its structure and folding was predicted by using the molecular graphics tool PyMOL.

### Immunoblotting

PBMCs were isolated from whole blood of the proband and the unaffected parents (family 5) by following the Mayo Clinic’s Biospecimen Accessioning and Processing (BAP) core’s standard protocol with a target count of 10 million cells. The isolated PBMCs were lysed in the buffer containing 150 mM NaCl, 50 mM NaF, 0.1% SDS, 1% NP40, 2 mM EDTA, 1% sodium deoxycholate, 1 mM DTT, 0.2 mM sodium orthovandate, and protease inhibitor (Roche) for 10 min on ice. The protein lysates were sonicated and centrifuged at 16,000 × *g* for 10 min to remove the debris. The protein concentration of each sample was determined by using Pierce BCA protein assay kit (Thermo-Scientific). Of the total protein, 40 µg was separated in 4%–12% NuPAGE gel (Novex/Life technologies) and transferred to a nitrocellulose membrane (Invitrogen). The membranes were blocked in 2.5% milk (BLOT- QuickBlocker™ EMD Millipore) in TBST followed by overnight incubation with primary antibody at 4°C. Membranes were washed, incubated with secondary antibody for 1 h at room temperature, and imaged on Odyssey® CLx (LI-COR Biosciences). The band intensities were measured using Image Studio™ (LI-COR Biosciences). All the primary and secondary antibodies were prepared in 2.5% blocking buffer.

The primary antibodies are as follows: Rabbit anti-GEMIN5 (1:1,000; Proteintech); Rabbit anti-GEMIN4 (1:2,000; Novas Biologicals); Rabbit anti-GEMIN2 (1:1,000; Invitrogen), Mouse anti-SMN (1:4,000; BD Biosciences); Mouse anti-α-tubulin (1:8,000; Sigma T5168).

The secondary antibodies are as follows: Goat anti-mouse Dylight 680 (1:10,000; LI-COR 925-68070); Goat anti-mouse Dylight 800 (1:10,000; Invitrogen SA5-10176); Goat anti-rabbit Dylight 680 (1:10,000; Invitrogen 35568); and Goat anti-rabbit Dylight 800 (1:10,000; Invitrogen SA5-35571).

### Knockout Mice Creation

Creation of the Gemin5 Knockout mice in the C57BL/6N background was carried out at the MRC Harwell Institute through the International Mouse Phenotyping Consortium (IMPC). Using CRISPR/Cas9 and the EUCOMM/KOMP-CSD allele structure, a premature stop codon was introduced into exon 7 of the *Mus muculus Gemin5* gene, resulting in a null *GEMIN5* allele. Heterozygous Gemin5 knockout mice [C57BL/6NTac-Gemin5em1(IMPC)H/H] were confirmed *via* short-range PCR at the MRC Harwell Institute.

## Results

### Identification of Bi-Allelic *GEMIN5* Variants

**FIGURE 1:**
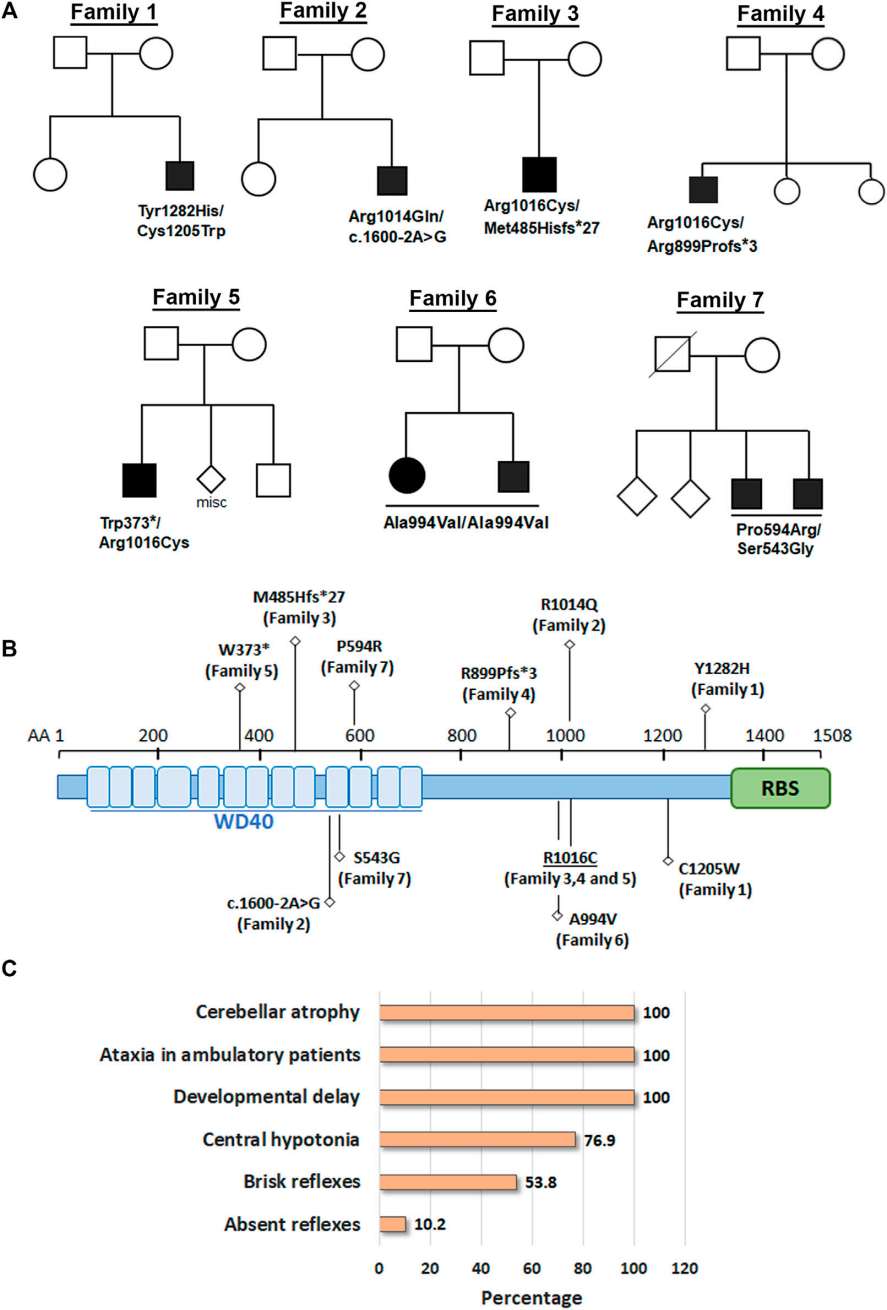
Biallelic variants in *GEMIN5* leads to cerebellar atrophy and spastic ataxia neurodevelopmental disease. **(A)** Pedigrees of seven unrelated families with nine affected individuals are shown in the top panel. Variants in *GEMIN5* are shown below the affected individuals. Affected individuals are shown as filled black circles/squires. **(B)** GEMIN5 protein domain structure. Type and position of the identified variants are shown along with the family numbers. **(C)** The frequency of clinical presentations in our patients with autosomal recessive variants in *GEMIN5*. The numerator and denominator in brackets indicate the number of affected probands and the number of probands assessed for the respective feature, respectively.

Here, we report nine patients with spastic ataxia and cerebellar atrophy (Figure 1). All patients had extensive metabolic and genetic testing, which was unrevealing before next-generation sequencing revealed biallelic variants in the *GEMIN5* gene (Table 1). All variants were rare with allele frequencies ranging from 0 to 4.48e−3 as a heterozygote and showed autosomal recessive inheritance pattern (Table 3). The majority of these variants, with two exceptions, (p. Arg1016Cys and p. Pro594Arg), have not been reported in the GnomAD database in the homozygous state (Table 3). The p. Arg1016Cys was observed with a second allele disrupted by either a frameshift or a nonsense variant in three patients from three unrelated families. The p. Arg1016Cys variant is likely to affect the dimerization domain, which might result in downstream functional abnormality. Similarly, we found the p. Pro594Arg allele with another missense variant in two patients (Table 1). The variants identified were missense, frame shift, termination, and a predicted splice site variant. All missense variants affected conserved amino acid residues and were predicted to be pathogenic by various computational prediction tools (Table 4).

**TABLE 1:** Clinical presentations in patients with biallelic variants in *GEMIN5* *.*

Family	1	2	3	4	5	6		7	
Patient number	1	2	3	4	5	6	7	8	9
Gender	M	M	M	M	M	M	F	M	M
Age of onset birth (B)	<1 year	<1 year	14 month	14 month	1 year	<1 year	<1 year	25 year	<1 year
Current age year (Y) Deceased (D)	18 month	5 Y	10 Y	12 Y	21 Y	21 Y	26 Y	60	57
Development									
Delayed? Yes: Y, No: N	Y	Y	Y	Y	Y	Y	Y	Y	Y
Regression Y/N	N	N	N	N	N	N	N	N	N
Motor delay Y/N	Y	Y	Y	Y	Y	Y	Y	N	Y
Speech delay Y/N/NA	Y	N	Y	Y	Y	Y	Y	Y	Y
Cognitive delay Y/N/NA; NA: not applicable	Y	N	Y	Y	Y	Y	Y	N	N
Neurological findings									
Ataxia Not walking (NW)	NW	Y	Y	Y	Y	Y	Y	Y	Y
Appendicular hypertonia Y/N	N	N	Y	N	N	N to mildly increased	Y	Y	Y
Central hypotonia Y/N	Y	N	Y	Y	Y	Y	N	N	N
Deep tendon reflexes Normal (N), brisk (B), absent (A)	N	N	B	N	B	B	B	B	B
Neurological evaluation									
Cerebellar atrophy (MRI) Yes (Y)/No (N)	Y	Y	Y	Y	Y	Y	Y	Y	Y
EMG/NCV				N				N	Mild sensorimotor neuropathy
Clinical course									
Static (S)/progressive (P)	S	S	S	S	P	S	S	S	P
Genetic variant	p.Tyr1282His. p.Cys1205Tryp	p. p.Arg1014Gln	p. Arg 1016cys p. Met485HisfsTer27	p.Arg1016Cys/ p.Arg899ProfsTer3	p.Trp373* mat/ P.Arg1016Cys pat	p.Ala994ValP.Ala994Val		p.Pro594Argp.Ser543Gly	

**TABLE 2:** Reanalysis of GEMIN5 variant (family 5).

#Chromosome	POS	ID	REF	ALT	QUAL	FILTER	INFO	FORMAT	Proband Family 5	Mother Family 5	Father Family 5
chr5	154305596	.	C	T	2350.16	PASS	AC = 2; AF = 0.333; AN = 6; BaseQRankSum = −2.172; ClippingRankSum = 0; DP = 273; ExcessHet = 3.9794; FS = 0.501; MLEAC = 2; MLEAF = 0.333; MQ = 60; MQRankSum = 0; QD = 12.91; ReadPosRankSum = 0.519; SOR = 0.644; VQSLOD = 6.58; culprit = MQRankSum	GT:AD:DP:GQ:PL	0/1:59,42:101:99:1144,0,1739	0/1:36,45:81:99:1238,0,1006	0/0:87,0:87:99:0,260,2936
chr5	154278839	.	G	A	4229.16	PASS	AC = 2; AF = 0.333; AN = 6; BaseQRankSum = −3.74; ClippingRankSum = 0; DP = 433; ExcessHet = 3.9794; FS = 1.131; MLEAC = 2; MLEAF = 0.333; MQ = 60; MQRankSum = 0; POSITIVE_TRAIN_SITE; QD = 13.96; ReadPosRankSum = 0.092; SOR = 0.811; VQSLOD = 8.22; culprit = MQRankSum	GT:AD:DP:GQ:PL	0/1:73,92:165:99:2449,0,2046	0/0:128,0:128:99:0,385,4375	0/1:65,73:138:99:1812,0,1654

**TABLE 3:** Allelic frequencies of the variants identified among our GEMIN5 patients.

Family	GEMIN5 variant	Prediction tool				
		PholyPhen-2	PROVEAN	SNAP2	mu PRO	SIFT
1	p. Tyr1282His	Damaging	Deleterious.	Pathogenic	−1.3449093 (DECREASE stability)	AFFECT PROTEIN function
	p. Cys1205Trp	Damaging	Neutral	Pathogenic	−1.6755575 (DECREASE stability	AFFECT PROTEIN function
2	p. Arg1014Gln	Damaging-	Deleterious-	Pathogenic-	−0.53089832 (DECREASE stability)	AFFECT PROTEIN function
	c. 1600-2A>G	—	—	—	—	—
3	p. Arg1016Cys	Damaging	Deleterious	Neutral	−0.96092825 (DECREASE stability)	AFFECT PROTEIN function
	p. Met485Hfs*27	Damaging	Neutral	Neutral	−1.4014936 (DECREASE stability)	AFFECT PROTEIN function
4	p. Arg1016Cys	Damaging	Deleterious	Neutral	−0.96092825 (DECREASE stability)	AFFECT PROTEIN function
	p. Arg899Pfs*3	Damaging	Deleterious	Pathogenic	−0.59989059 (DECREASE stability)	AFFECT PROTEIN function
5	p. Trp373*	—	—	—	—	—
	p. Arg1016Cys	Damaging	Deleterious	Neutral	−0.96092825 (DECREASE stability)	AFFECT PROTEIN function
6	p. Ala994Val	Damaging	Deleterious	Neutral	−0.031927059 (DECREASE stability)	AFFECT PROTEIN function
7	p. Pro594Arg	Damaging	Deleterious	Pathogenic	−0.49351856 (DECREASE stability)	AFFECT PROTEIN function
	p. Ser543Gly	Damaging	Deleterious	Pathogenic	−1.6477696 (DECREASE stability)	AFFECT PROTEIN function

**TABLE 4:** *In silico* prediction of *GEMIN5* variants.

Family	*GEMIN5* Variant (cDNA) (NM_015465.5)	*GEMIN5* Variant (protein) (NP_056280.2)	Allele frequency (heterozygous)	Number of homozygous
1	c.T3844C	p.Tyr1282His	4.01e−6	0
	c.C3615G	p.Cys1205Trp	0	0
2	c.3041G>A	p.Arg1014Gln	3.92e−5	0
	c.1600-2A>G	c.1600-2A>G	0	0
3	c.3026C>T	p.Arg1016Cys	4.48e−3	5
	c.1452dup	p.Met485HisfsTer27	0	0
4	c.3046 C>T	p.Arg1016Cys	4.48e−3	5
	c.2693-2700del	p.Arg899fsTer3	0	0
5	c.1119 G>A	p.Tyr373ter	0	0
	c.3046 C>T	p.Arg1016Cys	4.48e−3	5
6	c.2981C>T	p.Ala994Val	2.39e−5	0
7	c.1781 C>G	p.Pro594Arg	2.40e−3	6
	c.1624A>g	p.Ser543Gly	2.51e−4	0

**FIGURE 2:**
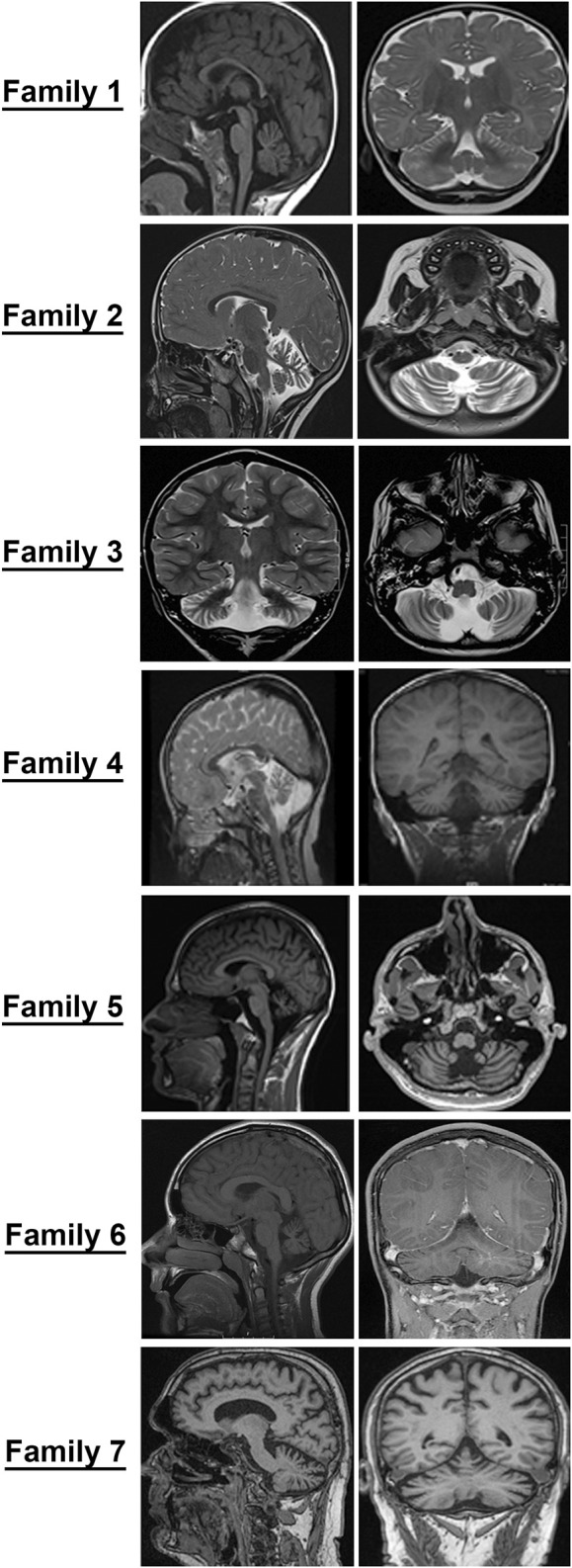
Cerebellar atrophy is a common feature among all individuals with *GEMIN5*-associated disease. Magnetic resonance imaging (MRI) of probands from families identified in the present study. Midsagittal, coronal, and axial T2/T1 images showing evidence of cerebellar atrophy, which was present in all patients.

Patients, 8/9, presented in the first 2 years of life with concerns for delayed motor development. No childhood motor or cognitive regression was observed in any of the patients. While all patients had signs of motor dysfunction, the neurological exam ranged from hypotonia and tremulousness to severe spastic ataxia. While central hypotonia was observed in some patients, appendicular tone ranged from normal to spastic. Interestingly, all patients presented with normal to brisk reflexes. All ambulatory patients were assessed by neurologists and were noted to be ataxic on clinical examination. The disease progression was variable among our patients, as some of the patients had a static course, while others showed a possibly slow, progressive ataxia. Magnetic resonance imaging (MRI) of the brain showed cerebellar atrophy in all patients (Figure 2).

Stratifying patients with GEMIN5-related disease from this study and our previously published cohort (Kour et al., 2021) by age of onset and severity revealed three main groups based on neurological involvement:

1. Infantile-onset severe global developmental delay with cerebellar atrophy on neuroimaging.

These patients presented in infancy with severe hypotonia and global developmental delay. Neuroimaging confirmed cerebellar atrophy.

2. Late infantile-onset developmental delay and ataxia syndrome with cerebellar atrophy on neuroimaging.
3. Juvenile/adult-onset spastic ataxia syndrome with cerebellar atrophy on neuroimaging.

Other clinical features seen in our patients include cataracts, strabismus, and nystagmus.

Overall, all variants of *GEMIN5* appear to perturb GEMIN5 structure and function(s) and result in deleterious neurological symptoms.

### Neuroradiological Features

The MRI images of all patients are shown in Figure 2. All patients showed diffuse cerebellar atrophy including vermian and hemispheric involvement of the cerebellum. Patient 5 had minimal brainstem and spinal atrophy as well. Most patients with repeat neuroimaging demonstrated a stable atrophy. At this time, it remains to be clarified if the loss of cerebellar volume is more of a hypoplasia rather than an atrophy and a natural history study with temporal follow-up of patients and imaging will be needed to better understand this.

### *GEMIN5* Variants Reduce the Levels of snRNP Complex Proteins in Patient Cells

**FIGURE 3:**
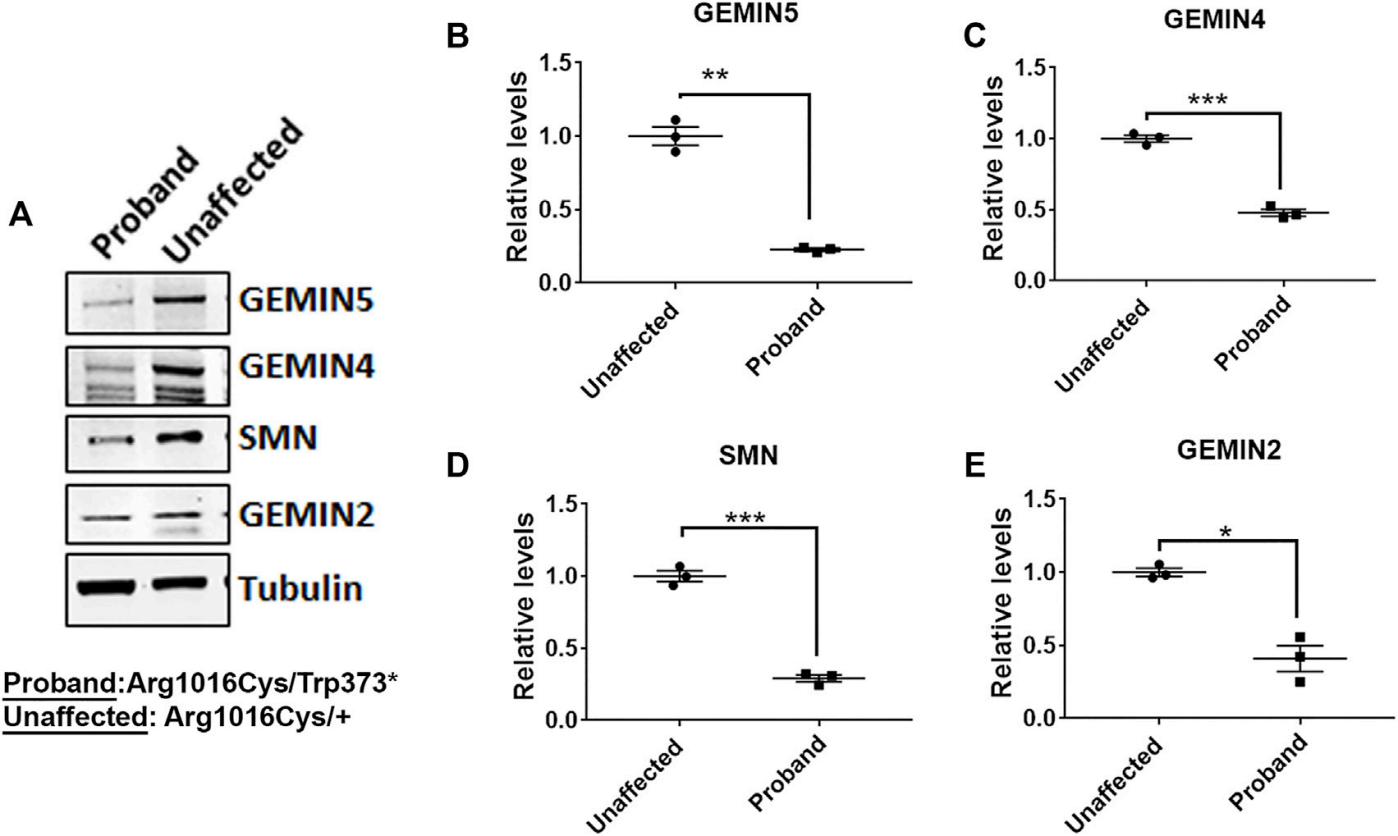
Individual with compound heterozygous variants in *GEMIN5* demonstrates reduced levels of SMN complex proteins. **(A)** Representative immunoblot showing the protein levels of SMN, GEMIN4, and GEMIN2 in the patient carrying p.Trp373* and p.Arg1016Cys variants in *GEMIN5*. Protein lysates were prepared from the PBMCs isolated from the patient and the unaffected parent (Arg1016Cys/+). **(B–E)** Quantitative plot showing the reduced levels of GEMIN5 **(B)**, GEMIN4 **(C)**, SMN **(D)**, and GEMIN2 **(E)** proteins in the patient relative to unaffected parent (father). The data represent mean ± SEM. *p*-Values (***<0.001, **<0.01, *<0.05) are calculated by unpaired *t* test (*n *= 3).

GEMIN5 is a multidomain protein, which acts as a crucial anchor during the assembly of small nuclear ribonucleoproteins (snRNPs), the essential components of the spliceosome (Pellizzoni et al., 2002; Wan et al., 2005; Battle et al., 2006a; Battle et al., 2006b; Kolb et al., 2007; Yong et al., 2010). We asked if the patients carrying compound heterozygous variants of *GEMIN5* perturb the levels of GEMIN5 and other SMN complex proteins. We isolated peripheral blood mononuclear cells (PBMCs) from patient 5 carrying p. Trp373* and p. Arg1016Cys variants of *GEMIN5*, as well as from the unaffected heterozygous parent (father) and performed Western blot (WB) with antibodies to GEMIN5, SMN, GEMIN4, and GEMIN2 to assess protein levels. We found a significant reduction in GEMIN5, SMN, GEMIN4, and GEMIN2 protein levels in the patient sample compared with the unaffected parent (Figure 3 and Supplementary Figure S2). These findings suggested that biallelic variants in *GEMIN5* result in deleterious changes in the levels of snRNP complex proteins, which is likely contributing to various neurological defects observed in our patients.

### *GEMIN5* Variants are Predicted to Cause Structural and Conformational Changes and Disrupt its Interaction With Other RNA/Proteins

**FIGURE 4:**
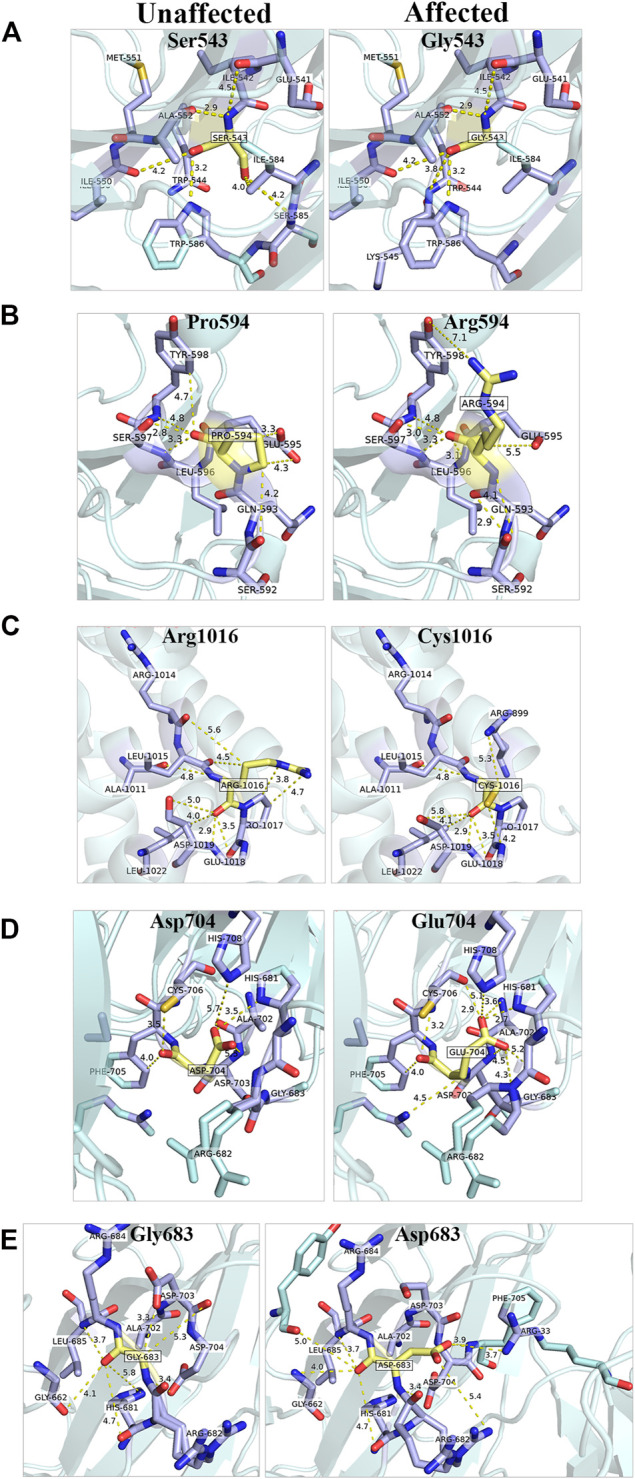
*In silico* prediction of structural changes caused by the *GEMIN5* variants. Structural and interaction-based changes were measured around 5A region of GEMIN5 variants Ser543Gly **(A)**, Pro594Arg **(B)**, Asp704Glu **(D)**, and Gly683Asp **(E)** in the WD40 domain (PDB ID: 5H1J) and Arg1016Cys **(C)** in the tetratricopeptide (TPR)-like domain (PDB ID: 6RNS) by using Pymol. The affected amino acids are colored yellow and their surrounding amino acid in light blue.

GEMIN5 is a highly conserved protein across vertebrates, and mutations in GEMIN5 have been shown to cause a loss of function and loss of protein stability (Kour et al., 2021) (Table 4). Various *in silico* tools allow us to predict the impact of an amino acid substitution on the structure and function of a human protein. Using *in silico* tools, we found that the *GEMIN5* variants are predicted to be deleterious in nature and lead to loss of protein function and stability (Table 4). Amino acid substitution at key sites within a protein due to a single-nucleotide variant (SNV) may result in various conformation changes, including remodeling or alteration of interaction network, salt bridges, and hydrogen bonds. These changes may perturb protein folding kinetics and can cause the destabilization of protein, impairing its subsequent function or interaction with other molecules (Dill et al., 1993; Teng et al., 2010). To determine the effect of *GEMIN5* missense variants on the protein conformation and interaction with surrounding amino acids, we used PyMOL to predict the structural changes caused by five possibly deleterious substitutions—three *GEMIN5* variants reported in our current study (p. Ser543Gly, p. Pro594Arg, and p. Arg1016Cys) and two *GEMIN5* variants from our previous study (p. Gly683Asp and p. Asp704Glu) (Kour et al., 2021). All of the following variants are located within the WD40 repeat domains (PDB ID: 5H1J), except p. Arg1016Cys, which is located in the tetratricopeptide (TPR)-like dimerization domain (PDB ID: 6RNS) of the GEMIN5 protein (Figure 4). By limiting the affected area of mutagenesis to 5 Å, we measured changes in the orientation and conformation of surrounding amino acid by calculating their distance from the substituted *GEMIN5* variant.

We found that the variant p. Ser543Gly in *GEMIN5* may lead to changes in its angular parameters and distance from Ile584, Ser585, and Lys545, respectively, which could result in structural changes in anaphase-promoting complex subunit 4, the WD40 domain (540–588 aa) of *GEMIN5* (Figure 4A). Similarly, we found that the conversion of Pro594, a nonpolar amino acid, to the positively charged arginine might affect its interactions with Glu595, Gln593, and Tyr598 (Figure 4B). Proline acts as a structural disruptor in alpha and beta helixes, which is a prerequisite for protein folding and structure. Therefore, its conversion to arginine, an amino acid found at the active centers of the protein and required for interactions with phosphorylated substrates, might result in the disruption of GEMIN5 structure and its interaction with other proteins.

PyMOL structure prediction suggested a possible change in the interaction and angular distance of 704Glu variant with His681, His708, Asp703, Gly683, and Arg682 (Figure 4D). Likewise, the p. Gly683Asp variant of *GEMIN5*, which is in a grove between two WD40 domains, induces an ionic charge that may result in the formation of additional bonds with Arg682 and Phe705 as well as change in the possible conformation due to its interaction with Arg33 (Figure 4E).

The p. Arg1016Cys variant of *GEMIN5* lies in the TPR-like dimerization domain, which modulates the interaction of GEMIN5 with other proteins. Thus, the substitution of the highly polar arginine, which is typically found in the interface of the two proteins, to a small and neutrally charged cysteine, may affect its conformation and dimerization function. PyMOL predicted the possible changes in the distance and bonding with Arg1014, Pro1017, and Asp1019 (Figure 4C). Furthermore, the *GEMIN5* variant p. Asp1019Cys has been reported to disrupt the dimerization properties of GEMIN5, suggesting the possible role and significance of Arg1016 and Asp1019 amino acids in protein–protein interactions. Overall, these findings suggest the possible deleterious effect of *GEMIN5* variants on its structure conformation, interaction, and function.

### Gemin5 is Essential for Development and Knocking out *Gemin5* Causes Embryonic Lethality in Mice

**FIGURE 5:**
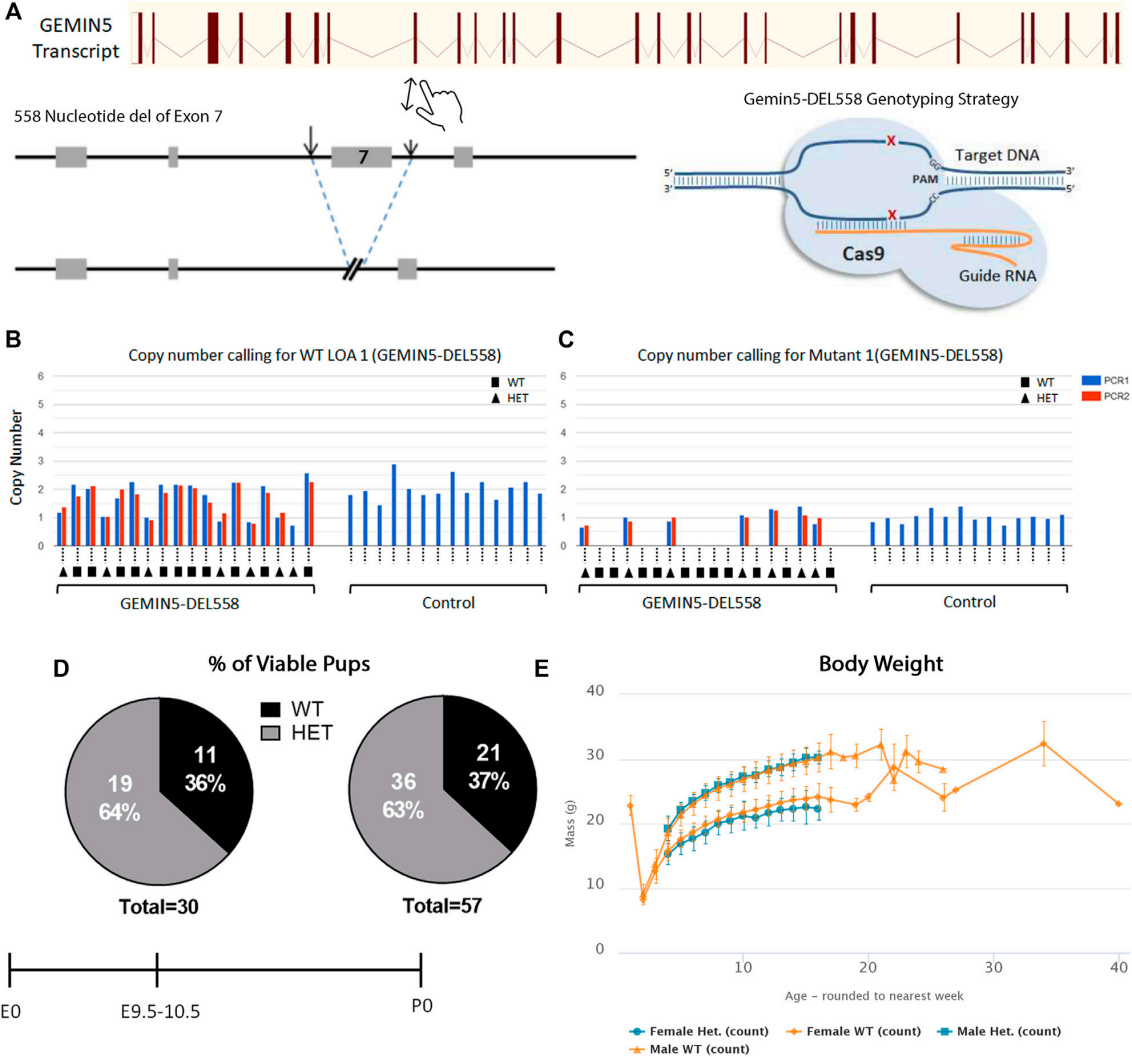
Knockout of Gemin5 leads to early embryonic lethality in mice. **(A)** Schematic representation of the wild-type allele of the mouse *Gemin5* transcript. The Gemin5 KO mouse (GEMIN5-DEL558) was created by utilizing the EUCOMM/KOMP-CSD allele structure to delete 558 nucleotides from exon 7 of the *Gemin5* gene, encompassing a critical exon to induce a premature stop codon and a null *Gemin5* allele (Mianné et al., 2017). **(B)** SR-PCR genotyping detects the presence of the Gemin5 wild-type allele by short-range PCR in Gemin5 KO and control mice (copy number of 2). Samples are genotyped with a WT loss of allele (WT-LOA) assay. This is a FAM-labeled assay that is designed to detect the critical exon that has been floxed. If the animal contains 1 Gemin5 KO allele, the copy number of this assay should drop by 1 (copy number of 1). For autosomal genes that have been targeted, this means the following: WT = 2 copies of the LOA assay, HET = 1 copy of the LOA assay, HOM = 0 copy of the LOA assay. **(C)** SR-PCR genotyping detects the presence of the cassette lacZ, the Gemin5 wild-type allele, and the Gemin5 KO allele confirming heterozygotes by short-range PCR (copy number of 1). **(D)** Viability of Gemin5 KO mice at the embryonic stage of E9.5 and as first-born pups at P0. Homozygous Gemin5 KO mice are not viable up to the embryonic stage of E9.5. **(E)** A body composition (DEXA lean/fat) phenotypic assay was performed on 1,028 mice. The charts show the results of measuring lean mass in 8 Het females and 7 Het male mice compared with 514 female and 499 male controls. Heterozygote mice appear to have no change in body mass compared with controls.

To further understand the consequences of loss of function in Gemin5 *in vivo*, Gemin5 knockout (GEMIN5-DEL558) mice were created through the International Mouse Phenotyping Consortium (Dickinson et al., 2016; Mianné et al., 2017). To successfully generate the Gemin5 knockout mice, CRISPR/Cas9 was utilized to delete 558 nucleotides including exon 7 of the *Mus musculus Gemin5* gene. This deletion of 558 nucleotides resulted in a frameshift and formation of a premature stop codon in the *Gemin5* gene (Figure 5A). Heterozygous mice were validated for harboring one copy of *Gemin5* WT and one copy of *Gemin5* knockout by SR-PCR genotyping (Figures 5B, C). Heterozygous knockout mice were crossed together to assess the percentage of homozygous pups. No postnatal homozygous pups were observed at the P0 stage. Of the P0 pups, 63% were heterozygous for the *Gemin5* knockout allele, while 37% of the pups harbored WT *Gemin5* (*n* = 57).

To assess if pups were viable during embryonic development, mice were genotyped at embryonic day E9.5–10.5 across five separate litters (Figure 5D). No homozygous embryos were observed at E9.5–10.5, suggesting that Gemin5 is crucial for embryonic development in mice (*n* = 30). In addition, the body weight of heterozygous Gemin5 knockout mice was assessed over the course of 20 weeks. There was no significant change in the weight of heterozygous mice compared with wild-type control mice (*n* = 15 heterozygote mice, 1,013 control mice) (Figure 5E). Since we did not get any Gemin5 null animals at E9.5, we were unable to determine the impact of abolishing Gemin5 on brain development. These findings suggest that the homozygous loss of Gemin5 results in early embryonic lethality, while harboring one copy of defective Gemin5 does not appear to be detrimental and cause any obvious motor defects (Supplementary Figure S3).

## Discussion

Here we expand on the neurological phenotypes observed among patients with biallelic pathogenic variants in the *GEMIN5* gene. The clinical spectrum ranges from global developmental delay of early infantile onset with cerebellar atrophy to adult-onset spastic ataxia syndrome with cerebellar atrophy. Hereditary ataxias are a genetically heterogenous group of disorders with more than 150 genes being associated with this phenotype to date. With our cohort, we define an autosomal recessive hereditary ataxia syndrome associated with biallelic variants in *GEMIN5.* All but our early infantile patients had normal to brisk reflexes suggesting pyramidal tract involvement. The adult patients in our cohort were primarily followed in a spastic paraplegia clinic.

Compiling the neurological phenotype from patients we previously reported (Kour et al., 2021) together with our current cohort, 21 of the currently reported 39 patients showed brisk reflexes, 4 had absent reflexes, 9 were normal, and 5 were unknown. Central hypotonia was seen in 30 of the 39 patients, 7 had normal central tone, and 2 were unknown. Of the 39 patients, 18 had appendicular hypertonia. Cerebellar atrophy was characteristically seen in all patients on neuroimaging, suggesting that this is a key feature associated with *GEMIN5* variants. We, hence, categorize the neurological phenotype associated with autosomal recessive variants in *GEMIN5* as predominantly in the hereditary cerebellar ataxia–spastic paraplegia spectrum of disease. While the hereditary cerebellar ataxias and hereditary spastic paraplegias have been thought to be clinically distinct, epidemiological studies and recent advances in next-generation sequencing technologies have demonstrated that multiple genes are associated with the disease phenotypes and overlaps in clinical presentation as well as molecular pathways.

Furthermore, this *GEMIN5* neurodevelopmental ataxia spectrum is characteristically distinct from the predominantly neuromuscular presentation of spinal muscular atrophy (SMA) caused by another member of the SMN protein complex. Of some overlap, pontocerebellar hypoplasia type 1 (PCH1) is a condition with pontocerebellar atrophy along with anterior horn cell involvement of the spinal cord associated with many autosomal recessive genes (Namavar et al., 2011; Wan et al., 2012; Rudnik-Schoneborn et al., 2013; Sánchez-Albisua et al., 2014; Lardelli et al., 2017; Ivanov et al., 2018; Nuovo et al., 2021). These diseases can present with a varying blend of neurological phenotypes including hypotonia to varying degrees of spasticity.

The *GEMIN5* variants identified, so far, are missense, deletion, frameshift, nonsense, and splicing defects with autosomal recessive inheritance pattern. The variants identified in our current study, with two exceptions (p. Arg1016Cys and p. Pro594Arg), were never reported in any publicly available databases. The p. Arg1016Cys variant was identified with a frameshift or nonsense variant in three patients from three unrelated families (Table 1). This variant has been reported with allelic frequency of 4.48e−3 (heterozygote), and there are five individuals reported as homozygous. It is believed that the p. Arg1016Cys variant is a hypomorphic variant, and it is not sufficient to manifest any clinical symptoms unless the other allele is fully disrupted. Alternatively, the homozygous individuals in gnomAD may be at a presymptomatic stage.

GEMIN5 is expressed predominantly in the cytoplasm as well as in the nucleus, nucleoplasm, and gem bodies, suggesting functions in multiple cellular compartments (Pacheco et al., 2009; Piñeiro et al., 2015; Francisco-Velilla et al., 2016; Lozano et al., 2018; Martinez-Salas et al., 2020; Moreno-Morcillo et al., 2020). Animal model studies suggest that complete or partial reduction of the snRNP complex proteins are detrimental (Saida et al., 2021). Knocking out *Smn*, *Gemin4*, and *Gemin5* is lethal in mice and *Drosophila*, suggesting that these proteins are essential for survival (Gates et al., 2004; Gavrilina et al., 2008; Meier et al., 2018). We have previously shown that genetic reduction of endogenous rigor mortis (fly homolog of human GEMIN5) leads to developmental delay, motor dysfunction, and reduced lifespan, similar to human patients with autosomal recessive variants in *GEMIN5* (Kour et al., 2021). Our data in human patient cells (Figure 3) suggest that pathogenic variants in *GEMIN5* significantly reduce the expression of snRNP complex assembly. Furthermore, knocking out *Gemin5* in mice is embryonically lethal (Figure 5). Although these findings are interesting, we are unable to establish a link between cerebellar atrophy and Gemin5 loss of function due to early embryonic lethality in our knockout Gemin5 mice. We performed *in silico* analysis to determine the impact of *GEMIN5* variants on the protein structure. We found that *GEMIN5* missense variants cause significant alterations on protein conformation, such as remodeling the interaction network, peptide chain bending, and the ability to interact with other amino acids. Since the GEMIN5 protein has been shown to dimerize, it is likely that frameshift and termination variants might perturb the kinetics of protein folding and can cause the destabilization of proteins. These alterations might disrupt the ability of GEMIN5 to form a complex with snRNP proteins and exert physiological functions, which in turn lead to neurological symptoms observed in patients with *GEMIN5*-related disease. We recently showed that reduction of the levels of GEMIN5 (by mutations or shRNA knockdown) is sufficient to disrupt the snRNP assembly in mammalian cells and iPSC neurons (Kour et al., 2021). These findings support the idea that each component of the snRNP complex has an important role to play in maintaining cellular functions. Alternatively, it is possible that loss of normal GEMIN5 function might affect translation of mRNAs either in a tissue-specific manner or ubiquitously leading to defective protein synthesis and their regulatory pathways.

Given the distinct neurological presentation, we propose the term *GEMIN5*-related neurodevelopmental ataxia with cerebellar atrophy to describe the variability in clinical presentation associated with this gene.

## Data Availability

The datasets presented in this article are not readily available due to patient-related privacy regulations and institutional policies. Requests to access the datasets should be directed to udai.pandey@chp.edu
