# Supplementary figures and images for: Autosomal Recessive Cerebellar Atrophy and Spastic Ataxia in Patients With Pathogenic Biallelic Variants in GEMIN5
Source: Front Cell Dev Biol. 2022 Feb 28;10:783762. doi: 10.3389/fcell.2022.783762 (PMC8918504; doi:10.3389/fcell.2022.783762)

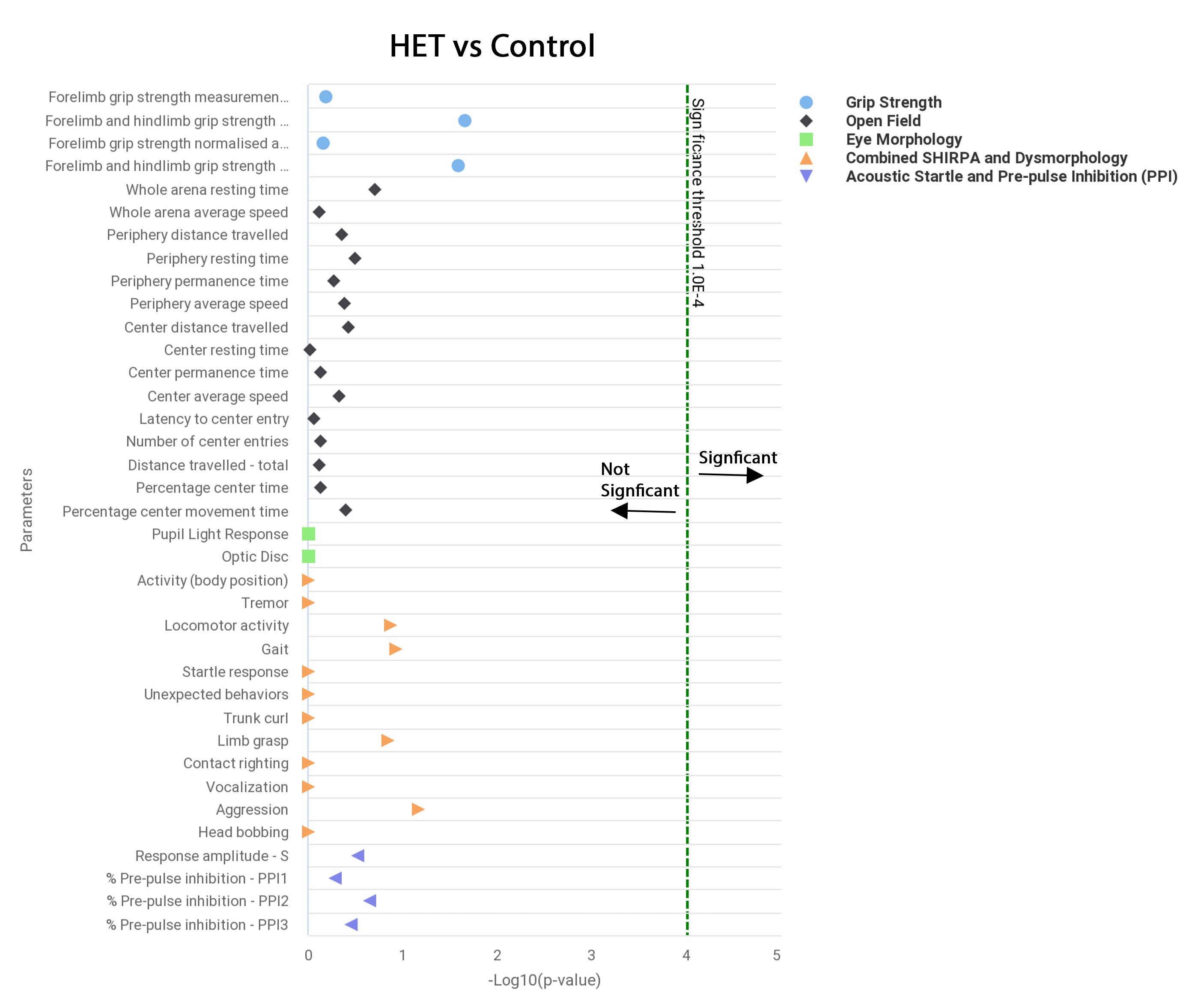

Supplement: Supplementary file 1 [file Image3.TIF]

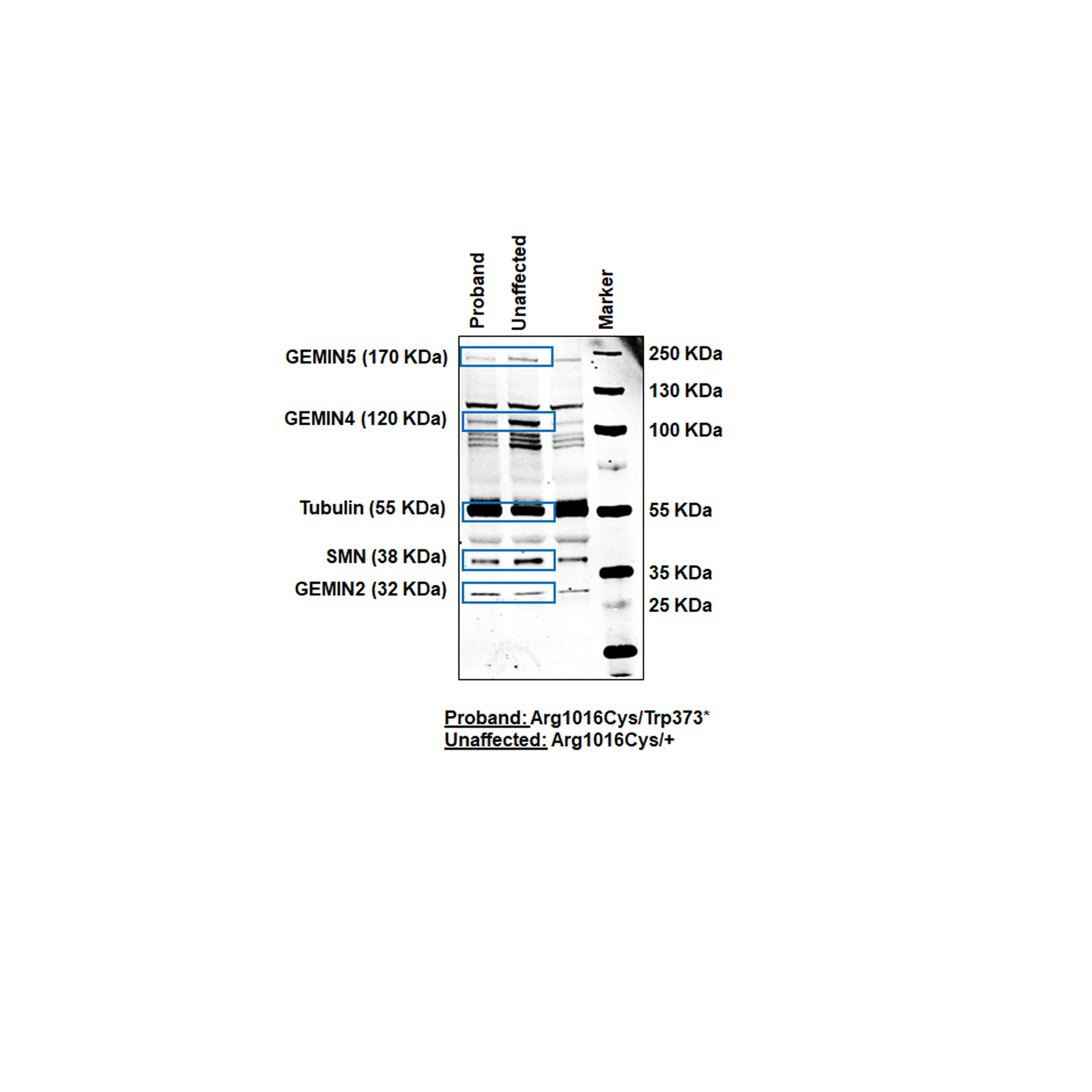

Supplement: Supplementary file 2 [file Image2.TIF]

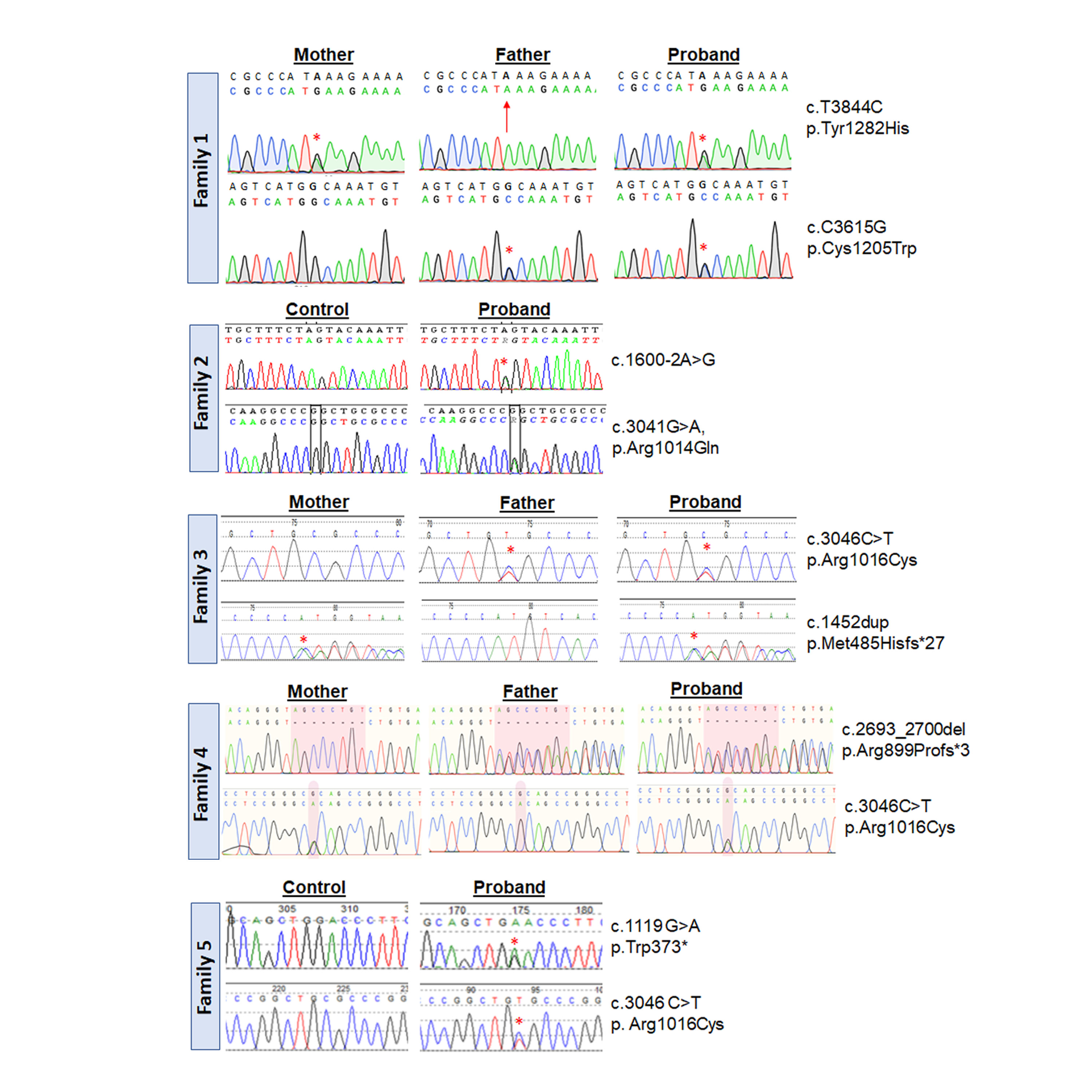

Supplement: Supplementary file 3 [file Image1.PNG]
